# Identifying the major lactate transporter of *Toxoplasma gondii* tachyzoites

**DOI:** 10.1038/s41598-021-86204-3

**Published:** 2021-03-24

**Authors:** Joy M. Zeng, Sanduni V. Hapuarachchi, Sarah H. Shafik, Rowena E. Martin, Kiaran Kirk, Giel G. van Dooren, Adele M. Lehane

**Affiliations:** Research School of Biology, Australian National University, Canberra, ACT 2601 Australia

**Keywords:** Parasite biology, Membrane proteins

## Abstract

*Toxoplasma gondii* and *Plasmodium falciparum* parasites both extrude l-lactate, a byproduct of glycolysis. The *P. falciparum* Formate Nitrite Transporter, *Pf*FNT, mediates l-lactate transport across the plasma membrane of *P. falciparum* parasites and has been validated as a drug target. The *T. gondii* genome encodes three FNTs that have been shown to transport l-lactate, and which are proposed to be the targets of several inhibitors of *T. gondii* proliferation. Here, we show that each of the *Tg*FNTs localize to the *T. gondii* plasma membrane and are capable of transporting l-lactate across it, with *Tg*FNT1 making the primary contribution to l-lactate transport during the disease-causing lytic cycle of the parasite. We use the *Xenopus* oocyte expression system to provide direct measurements of l-lactate transport via *Tg*FNT1. We undertake a genetic analysis of the importance of the *tgfnt* genes for parasite proliferation, and demonstrate that all three *tgfnt* genes can be disrupted individually and together without affecting the lytic cycle under in vitro culture conditions. Together, our experiments identify the major lactate transporter in the disease causing stage of *T. gondii*, and reveal that this transporter is not required for parasite proliferation, indicating that *Tg*FNTs are unlikely to be targets for anti-*Toxoplasma* drugs.

## Introduction

*Toxoplasma gondii* and *Plasmodium falciparum* are unicellular protozoan parasites that belong to the phylum Apicomplexa. *P. falciparum* is the most deadly of the *Plasmodium* species that cause malaria in humans^1^. *T. gondii* infects a large proportion of the world’s population and can cause severe disease in immunocompromised individuals^2^. *T. gondii* can also have devastating effects on the development of foetuses when it infects women during pregnancy^2^. New medicines are needed urgently against both parasites.

The disease-causing tachyzoite stage of *T. gondii* parasites utilizes both glucose and glutamine as energy sources for the generation of ATP^3,4^. These parasites acquire glucose through a plasma-membrane localized Glucose Transporter (*Tg*GT1)^3^. Glucose is metabolized via glycolysis to generate pyruvate. Pyruvate has multiple possible fates in the parasite^5^. It can be transported into the mitochondrion, where it is further metabolized by the TCA cycle to generate ATP by oxidative phosphorylation^6^. Alternatively, pyruvate can be metabolized to form l-lactate, which is then exported from the parasite^7,8^.

Lactate synthesis is catalyzed by lactate dehydrogenase (LDH). The *T. gondii* genome encodes two LDH enzymes (*Tg*LDH1 and *Tg*LDH2), neither of which is required for proliferation of the disease-causing tachyzoite stage under standard in vitro culture conditions^9,10^. *T. gondii* tachyzoites that are rendered defective in glucose import or some aspects of glycolysis can still grow in vitro and in vivo, catabolizing glutamine via the TCA cycle to produce ATP and generating essential glycolytic intermediates via gluconeogenesis^4,7,11–13^. When glucose and glutamine are both available, both are used for ATP generation^7^.

By contrast, the disease-causing blood stages of *P. falciparum* parasites rely almost exclusively on glycolysis to generate ATP^14^. Here, l-lactate is the major product of glycolysis and must be exported from the parasite in symport with H^+^ by the *P. falciparum* Formate Nitrite Transporter (*Pf*FNT) present on the parasite’s plasma membrane^15,16^. Members of the FNT family transport a variety of monocarboxylates and other anions^17–19^. They are found in numerous prokaryotic and eukaryotic microorganisms, but are not present in mammalian cells^15^. Compounds that kill *P. falciparum* parasites via inhibition of *Pf*FNT have recently been identified, thereby validating *Pf*FNT as a novel antimalarial drug target^20,21^.

A recent study provided evidence that the three homologues of *Pf*FNT encoded in the *T. gondii* genome are l-lactate transporters that localize to the plasma membrane when expressed from a non-native promoter^22^. Compounds found to inhibit the activity of the transporters showed some inhibition of tachyzoite proliferation at micromolar concentrations, leading to the suggestion that the *Tg*FNTs might be essential during the *T. gondii* lytic cycle and ‘druggable’^22^. The possibility that the compounds inhibited parasite growth via alternate means (‘off-target effects’) was not excluded. Although *T. gondii* tachyzoites can survive without glucose, it is possible that in the presence of glucose, the lack of a l-lactate efflux mechanism would cause l-lactate to accumulate intracellularly to concentrations sufficient to jeopardize pH regulation and/or osmotic stability. However, a genome-wide screen has provided evidence that each of the *Tg*FNTs are dispensable during the lytic cycle^23^, and a *Tg*FNT1 knockout parasite line was recently generated and found to grow normally despite secreting less lactate into the medium than wild-type parasites^24^. Here, we employed genetic and physiological approaches to investigate the roles of the three *Tg*FNTs in situ and to determine whether the activity of one or more *Tg*FNTs is required for the proliferation of *T. gondii* tachyzoites.

## Results

### *Tg*FNTs localize to the parasite plasma membrane

The *T. gondii* genome encodes three members of the FNT family, termed *Tg*FNT1 (TGGT1_209800), *Tg*FNT2 (TGGT1_292110) and *Tg*FNT3 (TGGT1_229170)^22^. The *Tg*FNTs share considerable sequence similarity with each other, with *Pf*FNT, and with the well-characterized *E. coli* FNT family member FocA (Supplementary Fig. S1).

**Figure 1:**
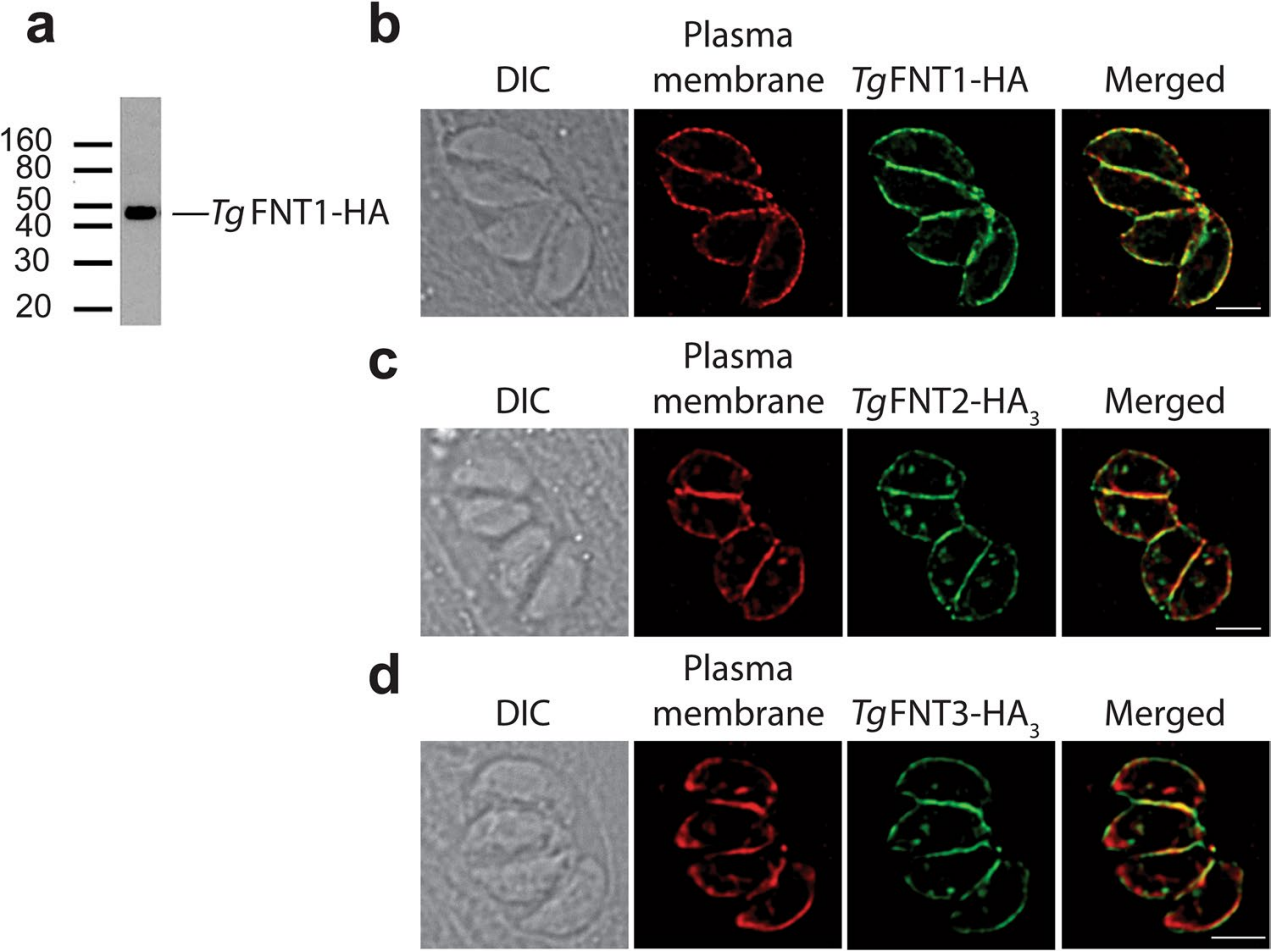
*Tg*FNT1 and ectopically-expressed *Tg*FNT2 and *Tg*FNT3 localize to the plasma membrane of *T. gondii*. (**a**) Western blot of *Tg*FNT1-HA-expressing parasites performed using an anti-HA antibody. The uncropped image is shown in Supplementary Fig. S3. (**b**–**d**) Immunofluorescence assay reveals co-localization of *Tg*FNT1-HA (**b**; green), *Tg*FNT2-HA_3_ (**c**; green) and *Tg*FNT3-HA_3_ (**d**; green) with the plasma membrane marker P30^39^ (red). The scale bars represent 2 µm.

To investigate the expression and localization of each *Tg*FNT, we attempted to generate parasite lines in which a single hemagglutinin (HA) tag was fused to the 3′ end of the open reading frame of the encoding gene. This approach was successful for *Tg*FNT1 (Supplementary Fig. S2). Western blotting with an anti-HA antibody revealed that the resultant *Tg*FNT1-HA protein is expressed in tachyzoites and that it had the expected mass of approximately 45 kDa (Fig. 1a). Immunofluorescence assays revealed that *Tg*FNT1-HA localizes to the *T. gondii* plasma membrane (Fig. 1b).

Our attempts to generate parasites expressing detectable levels of *Tg*FNT2-HA or *Tg*FNT3-HA with this approach were not successful. An alternate approach was therefore used to localize *Tg*FNT2 and *Tg*FNT3. Parasites were transiently transfected with a vector containing the open reading frame of the encoding gene fused at the 3′ end to a 3 × HA tag, under the regulation of the constitutive α-tubulin promoter. Consistent with the findings of Erler et al*.*^22^, the ectopically expressed *Tg*FNT2-HA_3_ and *Tg*FNT3-HA_3_ proteins both localized to the *T. gondii* plasma membrane (Fig. 1c,d).

### *Tg*FNTs are not required for tachyzoite proliferation in vitro

To determine whether any of the *Tg*FNTs are important for parasite proliferation, we used CRISPR-Cas9 targeted genome editing to generate parasites in which the open reading frame of the *tgfnt1*, *tgfnt2* or *tgfnt3* gene was disrupted by frameshift mutations. For *tgfnt1*, we selected a clone (‘Δ*tgfnt1*’) with a single nucleotide insertion at position 176 from the start codon of the gene. For *tgfnt2*, we selected a clone (‘Δ*tgfnt2*’) in which nucleotide 126 was deleted, and for *tgfnt3* (‘Δ*tgfnt3*’) we selected a clone with an insertion of five nucleotides from position 123 of the gene. In all cases, the resulting frameshifts led to the introduction of premature stop codons and truncation of the encoded proteins (Supplementary Fig. S4), which are likely to render the resulting proteins non-functional. We also generated a parasite line in which all three *tgfnt* genes were disrupted. This was performed by first targeting the *tgfnt1* gene in Δ*tgfnt3* parasites. We selected a clone (‘Δ*tgfnt1/3*’) that had a single nucleotide insertion at position 176 of the *tgfnt1* gene. Next, we targeted the *tgfnt2* gene in Δ*tgfnt1/3* parasites. We selected a clone (‘Δ*tgfnt1/2/3*’) with a single nucleotide insertion at position 127 of the *tgfnt2* gene. Each of the *tgfnt* genes in Δ*tgfnt1/2/3* parasites has a premature stop codon (Supplementary Fig. S4).

We tested whether any of the *Tg*FNT isoforms were important for parasite proliferation. We first assessed this using plaque assays. Plaques (zones of clearance) in a host cell monolayer result from multiple cycles of host cell invasion, replication and lysis, with the size of the plaques providing an indication of parasite growth rate. Δ*tgfnt1*, Δ*tgfnt2*, Δ*tgfnt3*, and Δ*tgfnt1/2/3* parasites all produced plaques comparable in size to those of their parents (Supplementary Fig. S5), indicating no significant growth defect in any of the four knockout lines.

**Figure 2:**
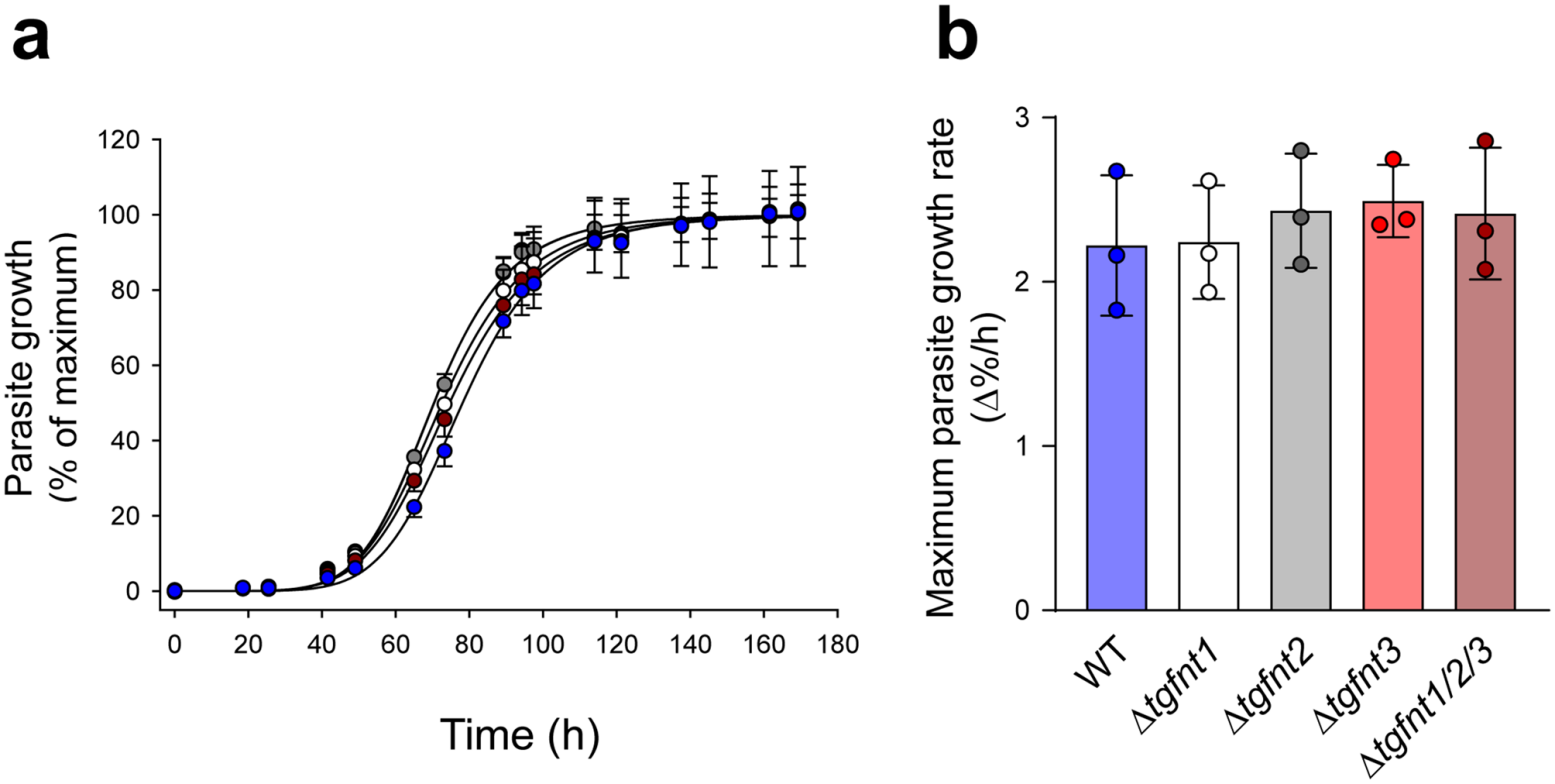
*Tg*FNTs are not required for tachyzoite proliferation in vitro. (**a**) Data from a single representative experiment showing the growth of the different parasite lines (wild-type, blue; Δ*tgfnt1*, white; Δ*tgfnt2*, grey; Δ*tgfnt3*, red; Δ*tgfnt1/2/3*, dark red). Parasite growth was normalized to the maximum level reached for each parasite line after subtraction of the background fluorescence. The mean and SD from triplicate measurements shown. Where not shown, error bars fall within the symbols. For clarity, only positive or negative error bars are shown for the data for certain parasite lines. (**b**) The maximum growth rate for each parasite line. The bars show the mean ± SD obtained from three independent experiments for each line, and the symbols show the results obtained in individual experiments. There was no significant difference in the maximum growth rate between any of the parasite lines (*P* = 0.8; one-way ANOVA).

We also compared the growth rates of the different parasites using a fluorescence assay (described previously^25^), taking advantage of the fact that all the parasites express a tandem dimeric Tomato (tdTomato) red fluorescent protein. In each experiment, a sigmoidal growth curve was generated for each parasite line, with a lag phase, exponential growth phase and plateau (Fig. 2a). Using the maximum slope of the growth curve as a read-out for parasite proliferation, we found no differences in growth rates among Δ*tgfnt1*, Δ*tgfnt2*, Δ*tgfnt3*, Δ*tgfnt1/2/3*, and wild-type parental parasites (Fig. 2b). We conclude that none of the *Tg*FNT isoforms, individually or collectively, are required for the normal proliferation of tachyzoites under in vitro culture conditions.

### *Tg*FNTs transport l-lactate, with *Tg*FNT1 making the primary contribution to l-lactate transport across the plasma membrane in extracellular tachyzoites

We used the *tgfnt* knockout parasites to investigate whether one or more of the *Tg*FNTs provide a route for l-lactate transport across the plasma membrane in *T. gondii*. The parasite experiments described in this section were all performed with extracellular tachyzoites that had either recently emerged from their host cells or that had been manually released from their host cells by passage of the cell culture through a 26G needle. First, we investigated the effect on parasite cytosolic pH of adding a high concentration of l-lactate to the extracellular solution. This approach has been used previously with *P. falciparum* parasites to investigate *Pf*FNT, which transports l-lactate in symport with H^+^ ions^15,16^. Although the physiological role of *Pf*FNT is to remove l-lactate from the parasite cytosol, it is bidirectional. Thus, when a high concentration of l-lactate is added to the extracellular solution to impose an inward l-lactate concentration gradient, a decrease in cytosolic pH resulting from l-lactate:H^+^ entry can be detected^15,21^.

**Figure 3:**
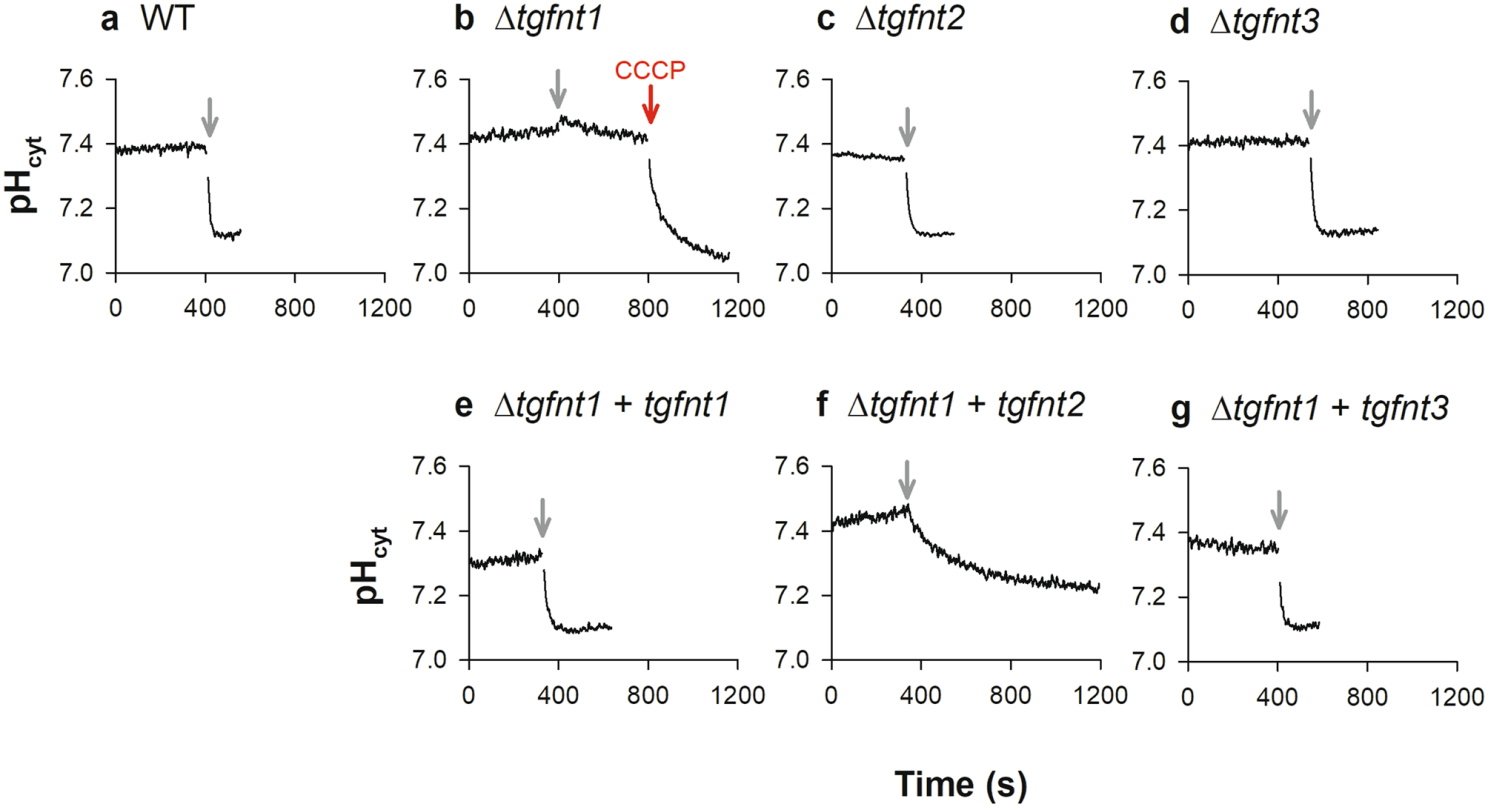
Measurements of the effect of l-lactate on cytosolic pH (pH_cyt_) reveal a role for *Tg*FNT1 in lactate transport across the parasite plasma membrane. Extracellular tachyzoites were loaded with BCECF and suspended in Saline Solution at 4 °C. The grey arrows denote the addition of 10 mM l-lactate. The red arrow denotes the addition of the protonophore CCCP (10 µM). The top panels show data for Δ*tgfnt1* (**b**), Δ*tgfnt2* (**c**), Δ*tgfnt3* (**d**) parasites and their wild-type parental parasites (**a**). The bottom panels show data for Δ*tgfnt1* parasites complemented with *tgfnt1* (**e**), *tgfnt2* (**f**) or *tgfnt3* (**g**). The traces are from a single experiment for each parasite line, and are representative of those obtained in at least three independent experiments. The time taken to reach a minimum pH_cyt_ value after the addition of l-lactate was > 400 s in all experiments for Δ*tgfnt1* parasites complemented with *tgfnt2*, compared to < 150 s in all experiments for WT parasites, Δ*tgfnt2* parasites, Δ*tgfnt3* parasites, and Δ*tgfnt1* parasites complemented with *tgfnt1* or *tgfnt3*.

We measured cytosolic pH in extracellular *T. gondii* tachyzoites by loading them with the pH-sensitive dye BCECF. The measurements were performed at 4 °C to reduce the rate at which pH-regulatory mechanism(s) (including the H^+^-extruding V-type H^+^ ATPase^26,27^) counteract the l-lactate-mediated pH change. When the wild-type parental parasites suspended in a physiological saline solution were exposed to 10 mM l-lactate, we observed a rapid decrease in the pH of the parasite cytosol (Fig. 3a). An abrupt l-lactate-induced decrease in cytosolic pH was also seen in Δ*tgfnt2* (Fig. 3c) and Δ*tgfnt3* (Fig. 3d) parasites. In contrast, the addition of 10 mM l-lactate to Δ*tgfnt1* parasites had little effect on the pH of the cytosol (Fig. 3b), consistent with parasites lacking *Tg*FNT1 having a greatly reduced capacity for l-lactate:H^+^ symport across the plasma membrane. The subsequent addition of the protonophore CCCP (10 µM) dissipated the H^+^ gradient across the plasma membrane, with the cytosolic pH decreasing to a value close to the extracellular pH (Fig. 3b), demonstrating the responsiveness of the pH-detection system in these cells.

To confirm that the reduction in l-lactate:H^+^ transport in Δ*tgfnt1* parasites was a consequence of the absence of *Tg*FNT1 expression, we complemented Δ*tgfnt1* parasites with an ectopically-expressed copy of *tgfnt1* (under the control of the constitutive α-tubulin promoter). In these parasites, the addition of 10 mM l-lactate to the extracellular solution caused a pronounced decrease in cytosolic pH (Fig. 3e). These data are consistent with *Tg*FNT1 serving as the primary l-lactate:H^+^ transporter on the plasma membrane in extracellular tachyzoites.

We also investigated whether *Tg*FNT2 or *Tg*FNT3 could restore l-lactate:H^+^ transport in Δ*tgfnt1* parasites when constitutively expressed in these parasites. We found that Δ*tgfnt1* parasites complemented with either *tgfnt2* or *tgfnt3* under the control of the α-tubulin promoter underwent a decrease in cytosolic pH on addition of 10 mM l-lactate to the extracellular solution (Fig. 3f,g). The rate of the l-lactate -induced cytosolic pH decrease was lower in Δ*tgfnt1* parasites complemented with *tgfnt2* (Fig. 3f) than in parasites complemented with *tgfnt1* (Fig. 3e) or *tgfnt3* (Fig. 3g). The initial rates (ΔpH/min, mean ± SEM, n = 3; determined by fitting lines to the initial linear portions of the traces) were 0.82 ± 0.19, 0.18 ± 0.09, and 0.57 ± 0.10 for Δ*tgfnt1* parasites complemented with *tgfnt1*, *tgfnt2* and *tgfnt3*, respectively. There was a significant difference between the rates of pH decrease for Δ*tgfnt1* parasites complemented with *tgfnt2* and Δ*tgfnt1* parasites complemented with either *tgfnt1* or *tgfnt3* (*P* < 0.05, unpaired t-tests). This could result from a difference between *Tg*FNT2 and the other *Tg*FNTs with respect to transporter function, regulation, and/or plasma membrane expression. Together, our data show that knockout of *Tg*FNT1 prevents l-lactate -induced pH changes in the parasite cytosol, which can be rescued by ectopic expression of *Tg*FNT1, *Tg*FNT2 and *Tg*FNT3. Our data also indicate that *Tg*FNT2 and *Tg*FNT3 make minimal contribution to l-lactate:H^+^ transport in wild-type tachyzoites. It is possible that the native *tgfnt2* and *tgfnt3* genes are not expressed at significant levels during this stage of the parasite life cycle.

**Figure 4:**
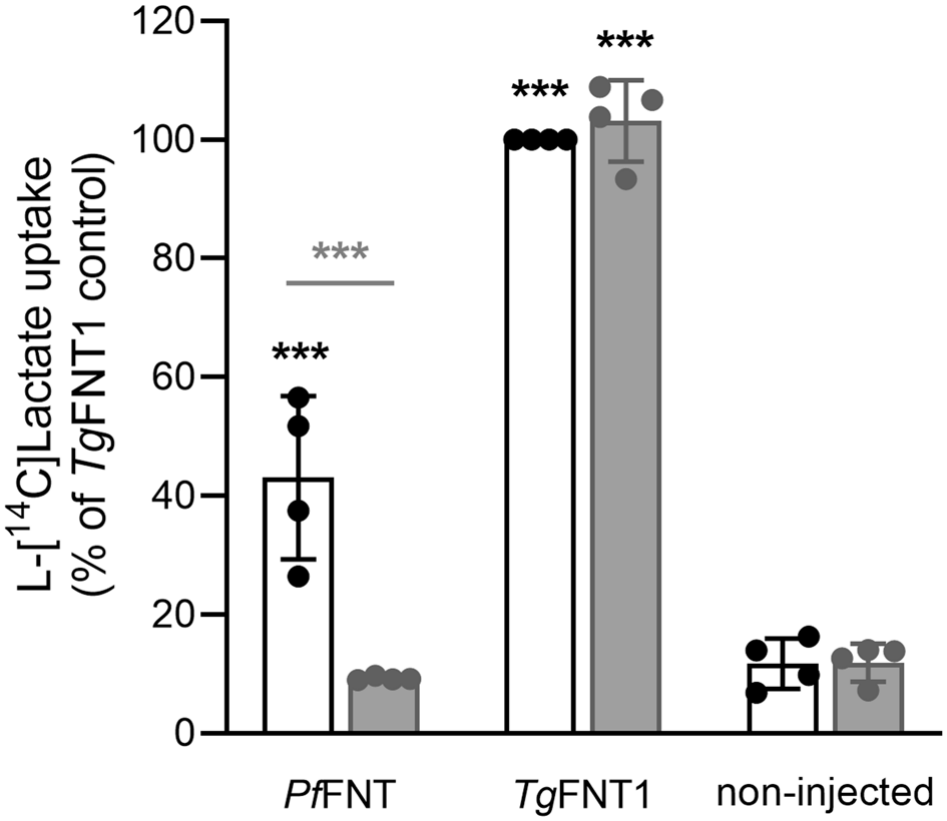
*Tg*FNT1 transports l-lactate in *Xenopus* oocytes. l-[^14^C]lactate uptake into non-injected oocytes and oocytes expressing *Pf*FNT or *Tg*FNT1-HA was measured in the absence of compound (0.008% v/v DMSO; solvent control; white bars) and in the presence of the *Pf*FNT inhibitor MMV007839 (4 µM; grey bars). The uptake of l-[^14^C]lactate is expressed as a percentage of that determined for *Tg*FNT1-HA in the absence of MMV007839. The data are averaged from four independent experiments (using oocytes from different frogs), within which measurements were made from 7 to 10 oocytes per treatment. The symbols show the data from each experiment and the bars show the mean ± SD. Where not shown, error bars fall within the symbols. The data were tested for statistical difference from the non-injected control (black asterisks). The data obtained in the presence or absence of MMV007839 were also compared for each oocyte type (grey asterisks). ***P < 0.001 (one-way ANOVA with post hoc Tukey test); other comparisons did not show significant differences.

Our pH data implicate *Tg*FNT1 as the primary l-lactate transporter in tachyzoites. Erler et al*.*^22^ provided evidence for l-lactate transport by *Tg*FNT1-3 expressed in yeast, however truncation of the proteins was required to achieve their functional expression. To test whether full-length *Tg*FNT1 is a l-lactate transporter, we expressed it in *Xenopus* oocytes. We have previously expressed *Pf*FNT in this system and shown that it transports l-[^14^C]lactate^15^ and is inhibited by the antiplasmodial compound MMV007839^21^. We measured the uptake of l-[^14^C]lactate into *Xenopus* oocytes expressing full-length *Tg*FNT1 (with a C-terminal HA tag) at 27.5 °C over 10 min at pH 6.4. The uptake of l-[^14^C]lactate by oocytes injected with 30 ng of cRNA encoding *Tg*FNT1 was higher than that of non-injected oocytes and of oocytes injected with 30 ng of cRNA encoding *Pf*FNT (*P* < 0.001; one-way ANOVA; Fig. 4). Consistent with previous results^21^, l-[^14^C]lactate uptake by *Pf*FNT-expressing oocytes was inhibited by MMV007839 (4 µM; Fig. 4). In contrast, 4 µM MMV007839 had no effect on the uptake of l-[^14^C]lactate by *Tg*FNT1-expressing oocytes (Fig. 4). These data provide direct evidence that *Tg*FNT1 is a l-lactate transporter. The finding that *Tg*FNT1 is less sensitive than *Pf*FNT to inhibition by MMV007839 is consistent with the results of Erler et al*.*^22^, who found that 29 µM MMV007839 was required for half-maximal inhibition of l-lactate transport by a C-terminally truncated form of *Tg*FNT1, whereas half-maximal inhibition of l-lactate transport by *Pf*FNT is achieved at sub-micromolar concentrations^20,21^.

**Figure 5:**
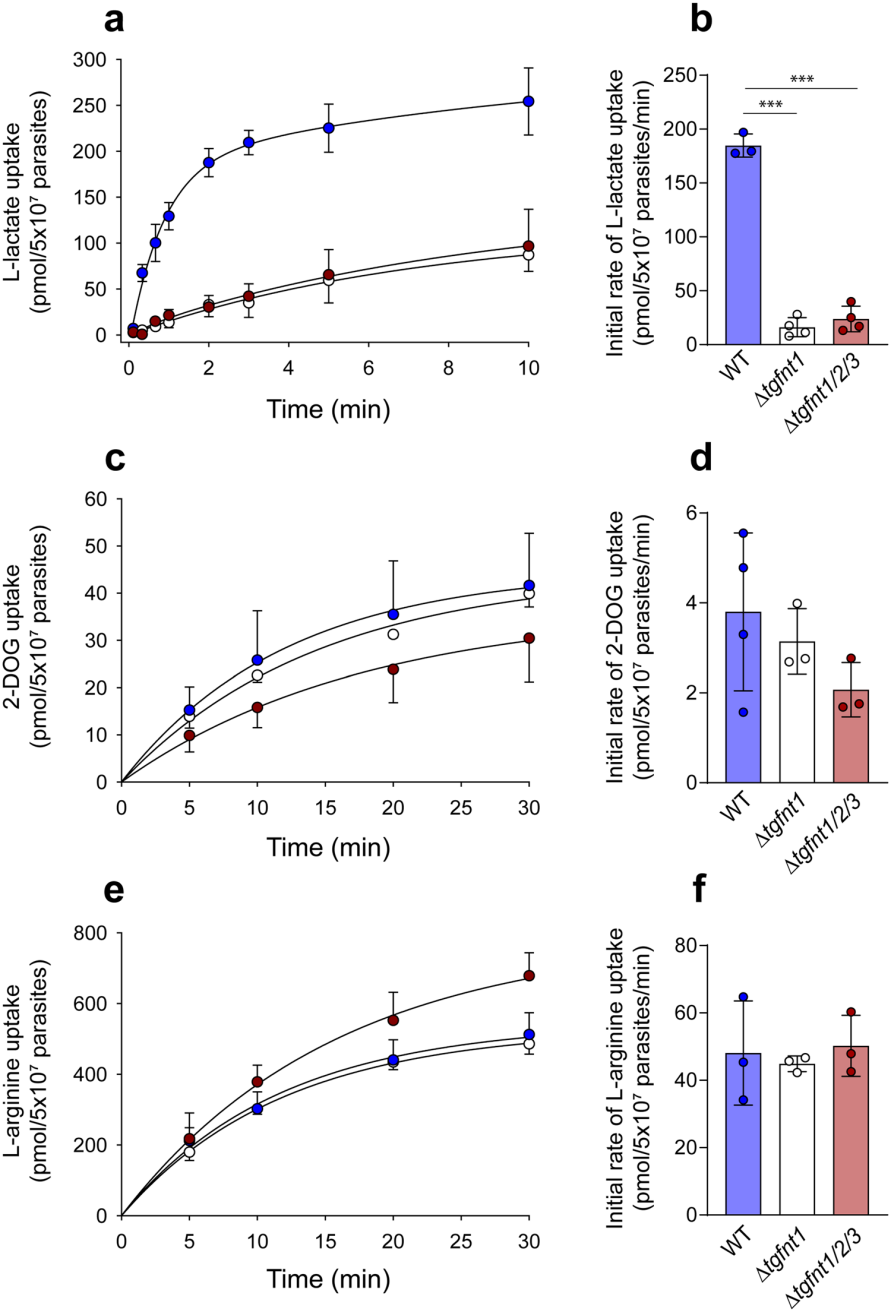
*Tg*FNT1 is critical for l-lactate transport across the parasite plasma membrane. (**a**,**c**,**e**) Time courses for the uptake of l-lactate (**a**), 2-DOG (**c**) and L-arginine (**e**) by wild-type parasites (blue symbols), Δ*tgfnt1* parasites (white symbols) and Δ*tgfnt1/2/*3 parasites (dark red symbols). The data shown are the mean ± SD from three to four independent experiments for each line. For clarity, only positive or negative error bars are shown for the data for certain parasite lines. Where not shown, error bars fall within the symbols. (**b**,**d**,**f**) The initial rates of uptake for l-lactate (**b**), 2-DOG (**d**), and L-arginine (**f**) estimated from the same set of experiments. The bars show the mean ± SD obtained from three or four independent experiments for each line, and the symbols show the results obtained in individual experiments. For panels **b**,**d** and **f**, the data for each parasite line were compared with the data for every other parasite line (one-way ANOVA, followed by post hoc Tukey tests where significant differences were present). ***P < 0.001; other comparisons (those in **d** and **f**) did not reveal significant differences.

As a final test of the role of *Tg*FNT1 in l-lactate transport across the plasma membrane in extracellular tachyzoites, we measured the uptake of l-[^14^C]lactate by extracellular Δ*tgfnt1*, Δ*tgfnt1/2/3*, and wild-type parental parasites. As noted above, FNTs mediate bidirectional transport, and measuring the movement of l-[^14^C]lactate *into* parasites provides a convenient means of assessing transport activity. Similar to previous experiments with *P. falciparum*^15,21^, the experiments with *T. gondii* presented here were conducted at a low pH (6.1) to increase (H^+^-coupled) l-lactate uptake by the parasites, and at a low temperature (4 °C) to slow the transport process. We found that the uptake of lactate, as measured over 10 min, was greatly reduced in Δ*tgfnt1* and Δ*tgfnt1/2/3* parasites relative to wild-type parental parasites, with little difference observed between Δ*tgfnt1* and Δ*tgfnt1/2/3* parasites (Fig. 5a). We estimated the initial rate of l-lactate uptake for each parasite line. The initial rate of l-lactate uptake was > 7.5-fold greater in wild-type parental parasites than in Δ*tgfnt1* or Δ*tgfnt1/2/3* parasites, and there was no significant difference in the initial rate between Δ*tgfnt1* and Δ*tgfnt1/2/3* parasites (Fig. 5b). Consistent with the pH data, these data indicate that *Tg*FNT1 plays the primary role in l-lactate transport across the plasma membrane in wild-type extracellular parasites.

To investigate the possibility that disruption of the *tgfnt1* gene could give rise to a general defect in solute uptake, we measured the uptake of two additional radiolabelled compounds: the glucose analogue [^14^C]2-deoxyglucose ([^14^C]2-DOG) and the amino acid l-[^14^C]arginine. The time courses for the uptake of 2-DOG and L-arginine were similar in wild-type, Δ*tgfnt1* and Δ*tgfnt1/2/3* parasites (Fig. 5c,e), and there were no significant differences in the initial rates of uptake for either of these compounds between any of the parasite lines (Fig. 5d,f). These data indicate that Δ*tgfnt1* and Δ*tgfnt1/2/3* parasites do not have a general defect in solute uptake, but rather have a specific defect in the uptake of FNT substrates including l-lactate.

## Discussion

In this study we pinpoint *Tg*FNT1 as the primary contributor to l-lactate transport across the plasma membrane of extracellular *T. gondii* tachyzoites. Tagging the 3′ end of the native *tgfnt1* gene revealed that the encoded protein localizes to the parasite plasma membrane. Experiments in which *Tg*FNT1 was studied in isolation in *Xenopus* oocytes provided direct evidence that full-length *Tg*FNT1 mediates the transport of l-[^14^C]lactate. Using genetic and physiological approaches we confirmed that *Tg*FNT1 functions as a plasma membrane l-lactate transporter in situ. We measured the rate of l-lactate transport across the parasite plasma membrane, and found that it was greatly reduced in Δ*tgfnt1* tachyzoites compared to wild-type parasites. We also investigated the effect on cytosolic pH of adding 10 mM l-lactate to the external medium. The decrease in cytosolic pH normally induced by a large inward l-lactate concentration gradient was not observed in Δ*tgfnt1* parasites, consistent with these parasites having a defect in l-lactate:H^+^ transport. Complementing Δ*tgfnt1* parasites with an ectopic copy of the *tgfnt1* gene restored the l-lactate-induced pH change.

Consistent with Erler et al.^22^, we found that *Tg*FNT2 and *Tg*FNT3 localize to the plasma membrane when expressed ectopically under the control of a non-native promoter. Furthermore, in line with previous evidence that truncated forms of *Tg*FNT1-3 transport l-lactate in yeast^22^, our data suggest that (full-length) *Tg*FNT2 and *Tg*FNT3 can transport l-lactate. Complementing Δ*tgfnt1* parasites with constitutively-expressed *tgfnt2* or *tgfnt3* resulted in the restoration of l-lactate:H^+^ transport, as observed in measurements of cytosolic pH.

Our data indicate that, despite being capable of transporting l-lactate, *Tg*FNT2 and *Tg*FNT3 make little contribution to l-lactate transport across the plasma membrane in wild-type extracellular tachyzoites. Disrupting *tgfnt1* alone was sufficient to abolish the l-lactate-induced acidification observed in parental parasites. Furthermore, there was no difference in the rate of l-[^14^C]lactate transport across the plasma membrane in Δ*tgfnt1* parasites and Δ*tgfnt1/2/3* parasites. Thus, expression of the endogenous *tgfnt2* or *tgfnt3* genes in Δ*tgfnt1* parasites does not appear to compensate for the l-lactate transport defect observed when *Tg*FNT1 is not expressed. It is possible that *Tg*FNT2 and *Tg*FNT3 are not expressed at appreciable levels during the lytic cycle in the *T. gondii* strain used in this study. This would also explain our inability to localize these proteins by tagging the 3′ ends of the endogenous genes. Our data are partially supported by a recent proteome-wide localization study of tachyzoites, which predicted that *Tg*FNT1 localizes to the plasma membrane whereas *Tg*FNT3 was not detectable in the proteome^28^. This study predicted that *Tg*FNT2 may localize to micronemes, which is inconsistent with our localization studies.

A key finding in our study was that the lack of expression of all three *Tg*FNT proteins does not affect the proliferation of *T. gondii* tachyzoites under standard in vitro culture conditions. This contrasts with the situation in *P. falciparum*, where *Pf*FNT has been validated as a drug target and must therefore be important for parasite proliferation^20,21^. However, it is consistent with previous studies highlighting the metabolic flexibility of *T. gondii* parasites, including recent evidence that the LDH enzymes required to convert pyruvate to lactate are not essential for *T. gondii* proliferation under standard in vitro culture conditions^9,10^. Our data are also consistent with a genome-wide screen that provided evidence that each of the *Tg*FNTs are dispensable during the lytic cycle (the ‘phenotype scores’ for *Tg*FNT1, *Tg*FNT2 and *Tg*FNT3 were 1.10, 1.31 and -0.23, respectively^23^), and with a very recent study in which *Tg*FNT1 was knocked out^24^. Kloehn et al*.*^24^ found that *Tg*FNT1 knockout parasites grew normally in vitro, but that they secreted less lactate into the medium than wild-type parasites. Our finding that all three *Tg*FNTs can be knocked out simultaneously call into question the view that the *Tg*FNTs are suitable drug targets, and suggest that the compounds reported to inhibit the activity of *Tg*FNT proteins by Erler et al*.*^22^ are unlikely to exert their effects on *T. gondii* growth via inhibition of any of the three transporters.

It should be noted that the disruption of certain glycolytic enzymes that are not essential for the in vitro proliferation of *T. gondii* parasites has been associated with virulence defects in mice and/or reductions in the formation of bradyzoite cysts in the brain. Parasites lacking hexokinase had a greatly reduced ability to form mature bradyzoite cysts in the brain in mice compared to parental parasites^13^. Furthermore, while tachyzoites in which the genes encoding *Tg*LDH1 and *Tg*LDH2 were simultaneously disrupted proliferated normally under standard conditions^9,10^, their growth was impaired in low-oxygen conditions in vitro^10^ and in mice^9,10^, and the numbers of bradyzoite cysts formed in the brain were greatly reduced^9,10^. Thus, it remains possible that the expression of one or more *Tg*FNT proteins is important in vivo and/or in different stages of the *T. gondii* life cycle.

## Methods

### Ethics statement

Ethical approval of the work performed with the adult female *Xenopus laevis* frogs was obtained from the Australian National University (ANU) Animal Experimentation Ethics Committee (Animal Ethics Protocol Number A2013/13) in accordance with the Australian Code of Practice for the Care and Use of Animals for Scientific Purposes.

### Host cell and parasite culture

*T. gondii* parasites were cultured in human foreskin fibroblasts (a gift from Holger Schlüter, Peter MacCallum Cancer Centre) at 37 °C in a humidified 5% CO_2_ incubator. The culture medium was Dulbecco’s modified Eagle’s medium (DMEM) containing 25 mM glucose, and supplemented with 1% v/v fetal calf serum, 200 µM L-glutamine, 50 U/mL penicillin, 50 µg/mL streptomycin, 10 µg/mL gentamicin, and 0.25 µg/mL amphotericin B. TATi/Tomato parasites^29^ were used as the parental wild-type strain for the ∆*tgfnt* parasite strains that we generated in this study. TATi∆*ku80* parasites^30^ were used as the parental wild-type strain for the 3′ replacement strains generated in this study.

### Generation of genetically modified *T. gondii* lines for localization studies

To generate a 3′ HA tag replacement in the *tgfnt1* locus, we amplified a 1.1 kb region of the 3′ end of the *tgfnt1* open reading frame using primers p4 and p5 (Supplementary Table S1). The PCR product was digested with *Bgl*II and *Avr*II and ligated into the *Bgl*II and *Avr*II sites of the vector pgCH(Δ*Pst*I), a variant of pgCH^31^ wherein the *Pst*I site has been filled in. The resulting vector was linearized with *Sph*I and transfected into TATi/Δ*ku80* parasites using standard procedures^32^, with successfully transfected parasites selected with chloramphenicol.

*Tg*FNT2 and *Tg*FNT3 were localized by expressing C-terminally 3 × HA tagged forms of the proteins under the control of the constitutive α-tubulin promoter. The *tgfnt2* open reading frame was amplified from *T. gondii* cDNA using primers p6 and p7 (Supplementary Table S1). The PCR product was digested with *Avr*II and *Bcl*I and ligated into the *Avr*II and *Bgl*II sites of the pUgCTH3 plasmid^31^. The *tgfnt3* open reading frame was amplified from cDNA using primers p8 and p9 (Supplementary Table S1). The PCR product was digested with *Avr*II and *Bgl*II and ligated into the *Avr*II and *Bgl*II sites of pUgCTH3. In both cases the vectors were transfected into TATi/tomato parasites^29^.

### Generation and complementation of *tgfnt* knockout parasites

We used a CRISPR/Cas9-based genome editing approach to generate parasite lines in which the open reading frames of *tgfnt* genes were disrupted. Using the Q5 Site-Directed Mutagenesis kit (New England Biolabs), we generated modified versions of the pSAG1::Cas9-U6::sgUPRT vector (Addgene plasmid # 54467^33^) to express guide RNAs targeting each *tgfnt* gene using a Q5 mutagenesis approach, as described by the manufacturer (New England Biolabs). The primers used for the Q5 mutagenesis reactions were p10 and p11 for *tgfnt1*, p12 and p11 for *tgfnt2*, and p13 and p11 for *tgfnt3* (Supplementary Table S1). The resulting vectors co-express the guide RNA targeting the appropriate *tgfnt* gene and Cas9-GFP. They were transfected into TATi/tomato parasites, generating Δ*tgfnt1*, Δ*tgfnt2*, and Δ*tgfnt3* parasites (Supplementary Fig. S4). Parasites in which two and three *tgfnt* genes were disrupted were then made by targeting the *tgfnt1* gene in Δ*tgfnt3* parasites then the *tgfnt2* gene in Δ*tgfnt1/3* parasites (Supplementary Fig. S4).

For complementation studies, Δ*tgfnt1* parasites were transfected with vectors containing the *tgfnt1*, *tgfnt2* or *tgfnt3* gene fused at the 3′ end to a 3 × HA tag, under the control of the constitutive α-tubulin promoter. For *Tg*FNT2 and *Tg*FNT3, the vectors used were those described above for the localization of the ectopically expressed proteins. The complementation vector for *Tg*FNT1 was made by amplifying the *tgfnt1* open reading frame from genomic DNA using primers p14 and p5 (Supplementary Table S1). The PCR product was digested with *Avr*II and *Bgl*II and ligated into the *Avr*II and *Bgl*II sites of pUgCTH3.

### Immunofluorescence assays and western blotting

Immunofluorescence assays (IFAs) and western blots were carried out as described previously^25^. The primary antibodies used were rat anti-HA (Roche clone 3F10; 1:100 – 1:1000 dilution; used for IFAs and western blots) and mouse anti-P30 (Abcam clone TP3; 1:500 dilution; used for IFAs). The secondary antibodies used for IFAs (all from Life Technologies) were goat anti-rat AlexaFluor 488 (1:100 – 1:250 dilution), goat anti-mouse AlexaFluor 546 (1:500 dilution), and goat anti-mouse AlexaFluor 647 (1:500 dilution). The microscopy was performed as described previously^26^. For western blots the secondary antibody was horse radish peroxidase-conjugated goat anti-rat (Santa Cruz Biotechnology; 1:5000 dilution).

### Parasite growth assays

Parasite growth was measured in the culture medium described above, which contains 25 mM glucose. Plaque assays were performed essentially as described previously^32^, with 400 parasites added per 25 cm^2^ flask. Fluorescence growth assays were performed in 96-well plates^25,34^, with 4000 parasites added to each well and the fluorescence from tdTomato (excitation: 540 nm; emission: 590 nm) used to monitor parasite growth. Prior to estimating parasite growth rates, a value corresponding to the background fluorescence (averaged from wells to which parasites had not been added) was subtracted from all other data. The fluorescence intensity corresponding to maximum growth was then determined for each parasite line by fitting a sigmoidal curve to the data: *y* = *a*/[1 + (*t*/*t*_1/2_)^b^], where *y* is fluorescence intensity, *a* is the maximum fluorescence intensity, *t* is time and *t*_1/2_ is the time at which half-maximal growth is reached. The data for each parasite line were then expressed as a % of the maximum growth for that line. For each parasite line, the maximum growth rate was determined from the slope of the sigmoidal curve in a 4 h window surrounding the *t*_1/2_ (estimated by fitting the sigmoidal curve described above).

### pH measurements in extracellular tachyzoites

Parasites were harvested by passage of cultures through a 26G needle. The parasites were then passed through a 3 µm filter to remove host cell debris and centrifuged for 20 min at 1500×*g*. The supernatant media was removed and the parasites were suspended in ‘BCF’ (bicarbonate-free RPMI supplemented with 25 mM HEPES, 11 mM additional glucose, 0.2 mM hypoxanthine and pH-adjusted to 7.1) then centrifuged at 12,000 × *g* for 1 min. The supernatant media was removed and the parasites suspended again in BCF, to which the acetoxymethyl ester (AM) form of the pH-sensitive dye BCECF (Molecular Probes) was added at a final concentration of 5 µM. The parasites were incubated in the BCECF-containing media for 14 min before being centrifuged (12,000 × *g*, 1 min), resuspended in BCF and maintained at 37 °C. Directly before their use in fluorescence measurements, parasites were centrifuged (12,000 × *g*, 1 min) and resuspended in pH 7.1 Saline Solution (125 mM NaCl, 5 mM KCl, 25 mM HEPES, 20 mM glucose and 1 mM MgCl_2_). Fluorescence measurements were performed at 4 °C in a Cary Eclipse Fluorescence Spectrophotometer (Agilent). The excitation wavelengths were 440 nm and 495 nm and the emission wavelength was 520 nm. The ratio of fluorescence (495 nm/440 nm) was used as an indicator of cytosolic pH. The relationship between Fluorescence Ratio and pH was determined by suspending parasites in calibration salines of known pH^35^ containing 16 µM nigericin (to equilibrate H^+^ across the plasma membrane) and measuring the Fluorescence Ratio after it stabilized.

### Measurements of the uptake of radiolabelled compounds by parasites

The uptake of radiolabelled compounds was measured in extracellular tachyzoites suspended in pH 6.1 Saline Solution (for l-[^14^C]lactate and l-[^14^C]arginine experiments) or Glucose-free Saline Solution (for [^14^C]2-DOG experiments; 125 mM NaCl, 5 mM KCl, 25 mM HEPES, 1 mM MgCl_2_; pH 6.1). The reactions for l-lactate experiments contained 200 µM unlabelled l-lactate and 0.2 µCi/mL l-[^14^C]lactate, those for the 2-DOG experiments contained 25 µM unlabelled 2-DOG and 0.2 µCi/mL [^14^C]2-DOG, and those for the L-arginine experiments contained 50 µM unlabelled L-arginine and 0.1 µCi/mL l-[^14^C]arginine.

The l-[^14^C]lactate and [^14^C]2-DOG experiments were conducted on ice, with centrifugation steps performed at 4 °C, to slow the rate of uptake such that initial rates could be determined. For the l-[^14^C]arginine experiments, parasites were incubated on ice for 30 min prior to commencing the experiments to mimic exposure conditions for the l-[^14^C]lactate experiments, but the experiments themselves were performed at 37 °C.

Uptake assays were performed as described previously^36^. To commence an experiment, a volume of parasite suspension was added to an equal volume of a saline solution containing the radiolabelled compound. The uptake of radiolabelled compounds was stopped at pre-determined time points (in duplicate 200 µL samples) by centrifuging the samples in tubes containing 250 µL oil mix (84% Clerco PM-125 silicone fluid, 16% light mineral oil), thereby sedimenting the parasites below the oil layer. Aliquots (10 µL) of the supernatant solution were taken from above the oil layer to enable the extracellular concentrations of the radiolabelled compounds to be determined. For each tube, the remaining supernatant solution was removed, the tubes washed twice in running water, the oil aspirated, and the parasite pellet lysed with 500 µL 0.1% v/v Triton X-100. The lysate was transferred into a scintillation vial containing 1.5 mL scintillation fluid (Ultima Gold; Perkin Elmer), and the radioactivity measured using a scintillation counter.

To account for the radioactivity present in the *extracellular* fluid trapped around the cell pellet under the oil layer, we subtracted the amount of radioactivity present at time zero from the remaining data. Where there was significant uptake of radioactivity early in the time course (l-[^14^C]lactate and l-[^14^C]arginine experiments), we fit a curve to the time course data for each experiment [*y* = *y*_*0*_ + *a*(1 – e^(-b*x*)^)] to determine the amount of radioactivity present at time zero (*y*_*0*_). For [^14^C]2-DOG experiments the radioactivity measured at the first time point (taken within 30 s of exposure of parasites to [^14^C]2-DOG) was subtracted directly. A curve [*y* = *a*(1 − e^(-b*x*)^)] was fitted to the data for intracellular radioactivity versus time and the initial rates were determined from the first derivative.

### Experiments with *Xenopus laevis* oocytes

The frogs were purchased from Nasco (Cat# LM00535M) and were housed in the *Xenopus* Frog Facility of the ANU Research School of Biology in compliance with the relevant institutional and Australian Government regulations.

The oocyte expression vector containing *pffnt* was made previously^15^. The oocyte expression vector containing *tgfnt1* was made by amplifying the *tgfnt1* open reading frame from gDNA using primers p14 and p5 (Supplementary Table S1). The PCR product was digested with *Avr*II and *Xma*I and ligated into the *Avr*II and *Xma*I sites of pGHJ-HA^31^.

Oocytes were harvested from *X. laevis* frogs and prepared as outlined previously^37^. cRNA encoding *Pf*FNT and *Tg*FNT1-HA was made using the mMessage mMachine T7 transcription kit and the MEGAclear kit (Ambion) and 30 ng was microinjected into oocytes, as described elsewhere^37^. The uptake of l-[^14^C]lactic acid (Na^+^ salt; 150.6 mCi/mmol; Perkin Elmer) was measured 3–4 days post-injection at 27.5 °C in ND96 buffer (96 mM NaCl, 2 mM KCl, 1 mM MgCl_2_, 1.8 mM CaCl_2_, 10 mM MES and 10 mM Tris-base; pH 6.4). Within each experiment measurements were made from 7 to 10 oocytes per condition. The influx of l-[^14^C]lactate was terminated by removing the reaction buffer and washing the oocytes twice in 3.5 mL of ice-cold ND96 buffer. The oocytes were then lysed and the radioactivity measured as described elsewhere^38^.

## Supplementary Information

Supplementary Information.
