# Supplementary material for: Identifying the major lactate transporter of Toxoplasma gondii tachyzoites
Source: Sci Rep. 2021 Mar 24;11:6787. doi: 10.1038/s41598-021-86204-3 (PMC7991638; doi:10.1038/s41598-021-86204-3)
Supplement: Supplementary file 1 — Supplementary Information. [file 41598_2021_86204_MOESM1_ESM.pdf]

Identifying the major lactate transporter of *Toxoplasma gondii* tachyzoites

Joy M. Zeng, Sanduni V. Hapuarachchi, Sarah H. Shafik, Rowena E. Martin, Kiaran Kirk, Giel G. van Dooren¶* and Adele M. Lehane¶*

Research School of Biology, Australian National University, Canberra, ACT, Australia

¶Joint senior authors

*To whom correspondence should be addressed: Giel G. van Dooren: Research School of Biology, Australian National University, Canberra, ACT 2601; giel.vandooren@anu.edu.au; Tel. +61 2 6125 0665; and Adele M. Lehane: Research School of Biology, Australian National University, Canberra, ACT 2601; adele.lehane@anu.edu.au; Tel. +61 2 6125 6970

Supplementary Information

Included material:

Supplementary Table S1. Primers used in this study.

Supplementary Figure S1. Multiple protein sequence alignment of *Tg*FNT1, *Tg*FNT2, *Tg*FNT3, *Pf*FNT and *E. coli* FocA (*Ec*FocA).

Supplementary Figure S2. Generation of *Tg*FNT1-HA parasites.

Supplementary Figure S3. Uncropped western blot of *Tg*FNT1-HA.

Supplementary Figure S4. Disruption of the *tgfnt* genes using CRISPR-Cas9 targeted genome editing.

Supplementary Figure S5. Plaques produced by *tgfnt* knockout parasites and their wild-type parents.

Supplementary Table S1. Primers used in this study.

p1	GACTACGGTTTGAAGAAGGTGC
p2	GACCTAAGCCCTCTCCCGA
p3	GTCATCCCTTTTCTTCGATAA
p4	ctgaagatctGAACGCATGTATTTCGTTGGC
p5	gactcctaggGCTTGTCGCTGTGGGCG
p6	gatctgatcaaaaATGTGCTCGATACCGCCACTACG
p7	gatccctaggCACTTGCTGGGCCACCG
p8	gatcagatctaaaATGGTGCTTGCGGCCAGTCCTGAGG
p9	gatccctaggAATGGTAGACGCCGGGTGCTCTTCAAGTG
p10	CGTGATCGCCTTCGGGTCCGGTTTTAGAGCTAGAAATAGCAAG
p11	AACTTGACATCCCCATTTAC
p12	GAAGTGAATTCCTTCCTCATGTTTTAGAGCTAGAAATAGCAAG
p13	GGAATATACGTCGGCATGGCGTTTTAGAGCTAGAAATAGCAAG
p14	gatcagatctcccgggccaccATGGTaGTGACAGCGTCTCCG

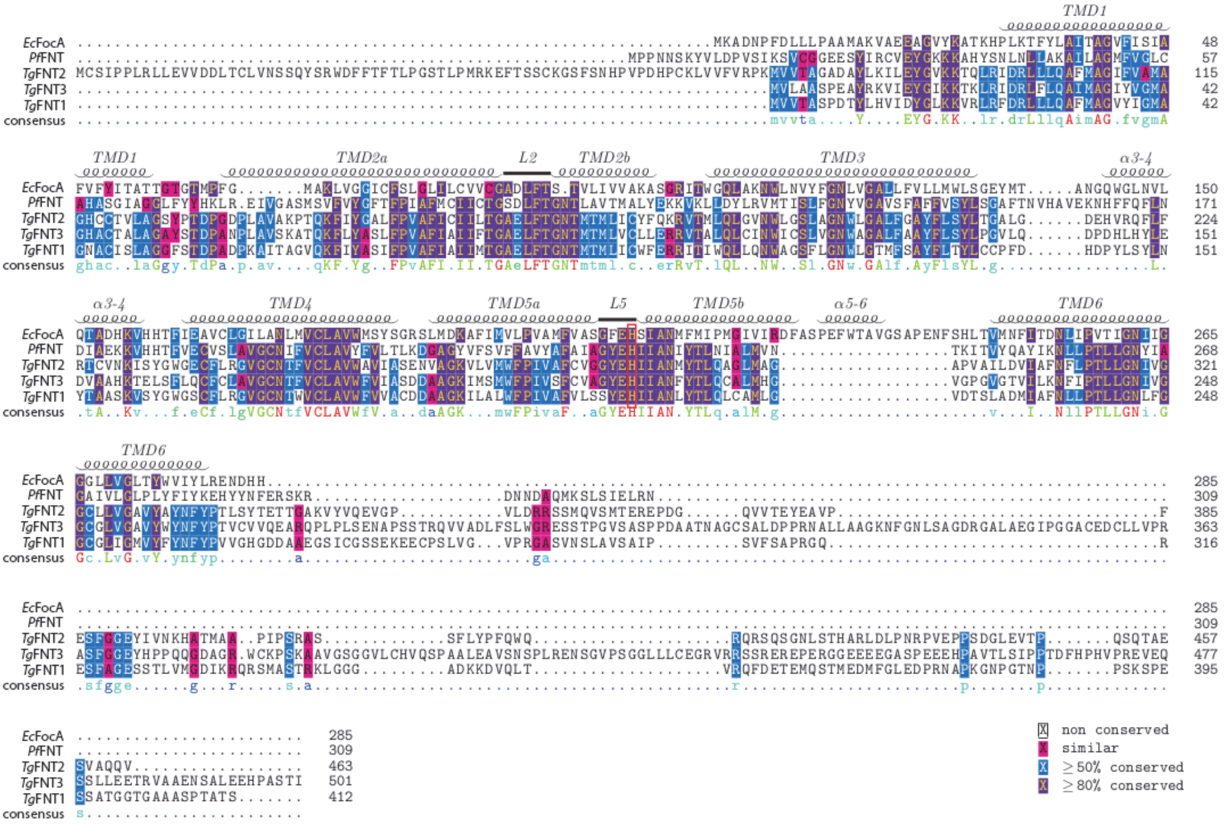

Supplementary Figure S1. Multiple protein sequence alignment of *Tg*FNT1, *Tg*FNT2, *Tg*FNT3, *Pf*FNT and *E. coli* FocA (*Ec*FocA). The six predicted transmembrane domains[1] are marked with "TMD", and numbered sequentially. The positions of the helix-interrupting loops L2 and L5 are marked by bars. Two additional alpha helices connect TMD3 and TMD4 (α3-4), and TMD5 and TMD6 (α5-6) of FocA. The invariant histidine residue (TMD5) found in all characterized FNT family proteins is boxed in red. The consensus sequence is shown; lower case residues indicate 50-79% conservation, upper case residues indicate ≥80% conservation. The alignment was generated using ClustalX2, and annotated using TeXshade[2].

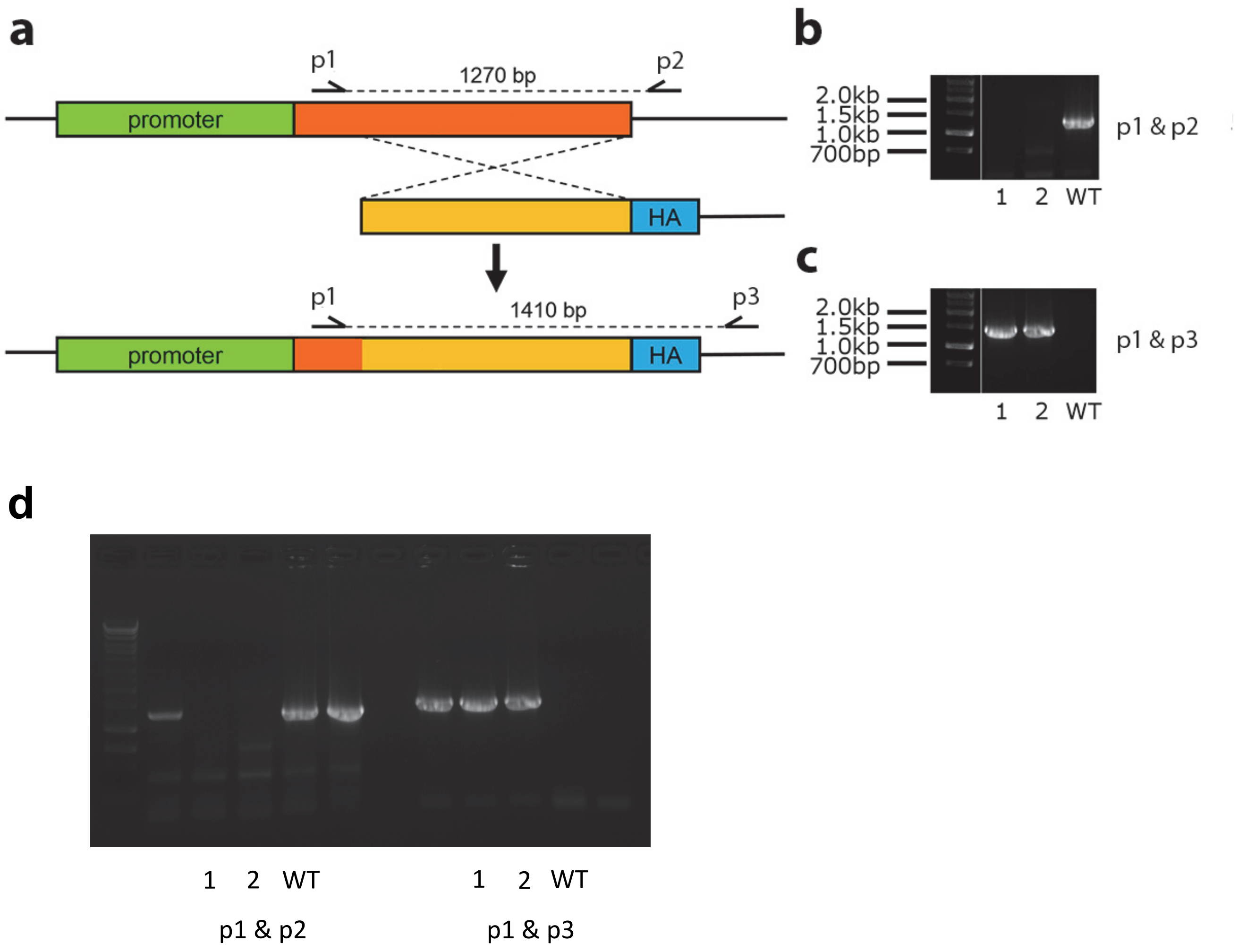

Supplementary Figure S2. Generation of *Tg*FNT1-HA parasites. (**a**) *tgfnt1* is under the control of its native promoter (green). The 3' replacement sequence (light orange) with an HA tag (blue) fused to the 3' end integrates into the native locus (dark orange) by single crossover homologous recombination, thereby introducing the HA tag to the 3' end of the open reading frame of the gene. Two PCR screens were performed to confirm the successful integration of the 3' replacement sequence: one with a primer combination (p1 and p2; Supplementary Table S1) that would only produce a specific band if there had been no change to the native locus (**b**), and one with a primer combination (p1 and p3; Supplementary Table S1) that would only produce a band if the integration event shown in panel **a** had occurred (**c**). The WT PCR reactions were performed with the TATi/*Δku80* parent line; reactions 1 and 2 were performed with separate clones of *Tg*FNT1-HA. (**d**) The full-length gel from which the cropped images shown in panels **b** and **c** were obtained, with the lanes used in panels **b** and **c** indicated.

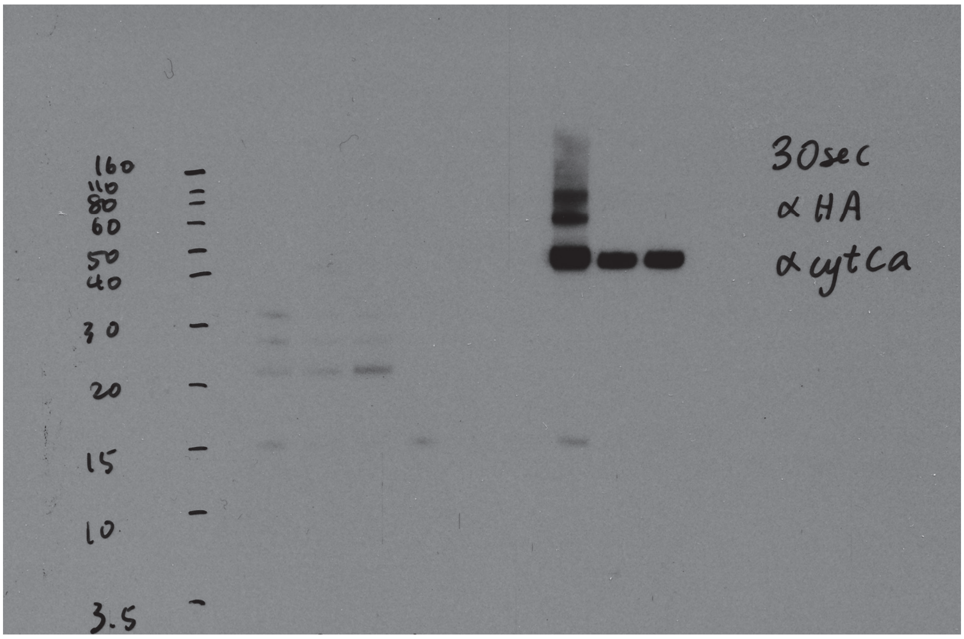

*

Supplementary Figure S3. Uncropped western blot of *Tg*FNT1-HA. The image shown in Fig. 1a is from the lane denoted by the asterisk, in which an anti-HA antibody was used to detect the *Tg*FNT1-HA protein in a protein sample prepared from a clonal *Tg*FNT1-HA line.

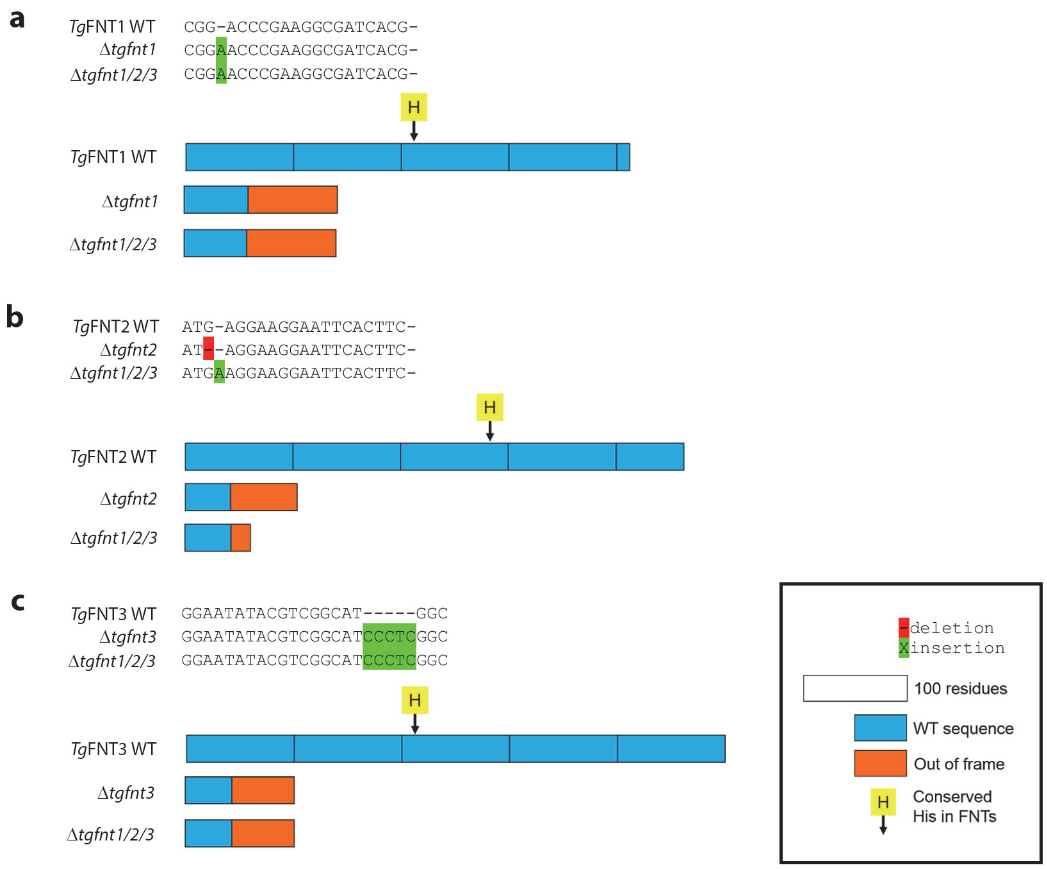

Supplementary Figure S4. Disruption of the *tgfnt* genes using CRISPR-Cas9 targeted genome editing. The modifications to the *tgfnt1* gene (**a**), *tgfnt2* gene (**b**) and *tgfnt3* gene (**c**) in the *tgfnt* knockout lines characterized in this study are shown. The schematics illustrate the resulting proteins expected from the modified genes, with in-frame (blue) and out-of-frame (orange) regions of the protein shown. The schematics are drawn to scale, with each large rectangle representing 100 residues. All of the modifications resulted in frameshifts and premature stop codons in the genes, which (if expressed) would give rise to truncated proteins lacking the critical histidine residue conserved in FNT family members.

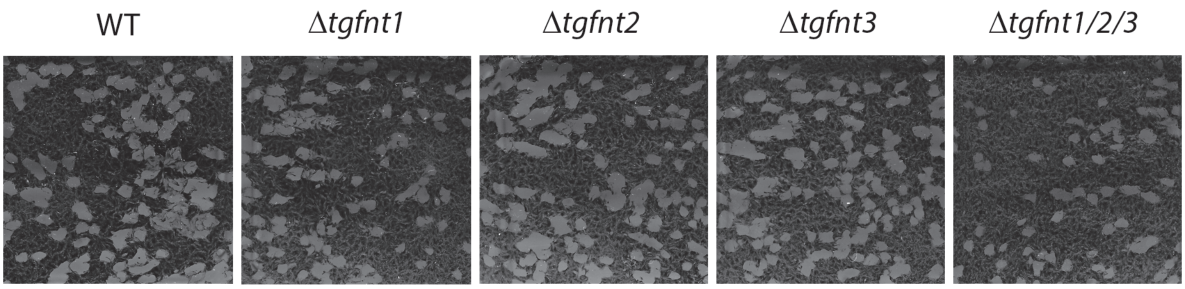

Supplementary Figure S5. Plaques produced by *tgfnt* knockout parasites and their wild-type parents. The images were scanned from host cell monolayers, which were stained with crystal violet after a 10 day period of parasite growth.

References

1. Wang, Y., Huang, Y., Wang, J., Cheng, C., Huang, W. *et al.* Structure of the formate transporter FocA reveals a pentameric aquaporin-like channel. *Nature* **462**, 467-472 (2009).
2. Beitz, E. TEXshade: shading and labeling of multiple sequence alignments using LATEX2 epsilon. *Bioinformatics* **16**, 135-139 (2000).
